# (20S) Ginsenoside Rh2-Activated, Distinct Apoptosis Pathways in Highly and Poorly Differentiated Human Esophageal Cancer Cells

**DOI:** 10.3390/molecules27175602

**Published:** 2022-08-31

**Authors:** He Li, Chunxiao Han, Chen Chen, Guanghong Han, Yang Li

**Affiliations:** 1 Key Laboratory for Molecular Enzymology and Engineering, The Ministry of Education, College of Life Science, Jilin University, Changchun 130012, China; 2 Changchun Institute of Biological Products Co., Ltd., Changchun 130012, China; 3 Ningbo Women and Children’s Hospital, Ningbo 315000, China; 4 Department of Oral Geriatrics, Hospital of Stomatology, Changchun University, Changchun 130012, China

**Keywords:** apoptosis, caspase, death receptor, esophageal cancer, ginsenoside Rh2, mitochondria-mediated apoptosis pathway

## Abstract

Ginsenoside Rh2 (G-Rh2), a rare ginsenoside isolated from red ginseng, has considerable anti-cancer activity and induces apoptosis in a variety of cancer cells, but its activity in esophageal cancer cells is unclear. In this study, we examined the cytotoxic activity of (20S) G-Rh2 in highly differentiated esophageal squamous ECA109 cells and poorly differentiated esophageal squamous TE-13 cells. (20S) G-Rh2 exerted intense cytotoxicity in ECA109 and TE-13 cells with an IC50 of 2.9 and 3.7 μg/mL, respectively. After treatment with G-Rh2, Bcl-2, and Bcl-xL, the two main anti-apoptosis Bcl-2 family proteins upregulated, and Bax and Bak, the two key pro-apoptosis proteins translocated to mitochondria in both cell lines. At the same time, cytochrome *c* and Smac released from mitochondria, followed by caspase-9 activation, indicating that a mitochondria-mediated intrinsic apoptosis pathway was activated in both cell lines upon treatment with (20S) G-Rh2. It is noteworthy that (20S) G-Rh2 upregulated the transcription and protein expression of two death receptors, Fas and DR5, and subsequently activated Caspase-8 in the TE-13 cells but not in the ECA109 cells. Taken together, we demonstrated the potent anti-esophageal cancer cell activity of (20S) G-Rh2 and showed its working mechanism in two differentiated esophageal cancer cells, which can provide important evidence for developing an effective strategy for anti-esophageal cancer treatment.

## 1. Introduction

Esophageal cancer is one of the most malignant tumors in the world, which can be divided into squamous cell carcinoma and adenocarcinoma [1]. Esophageal squamous cell carcinoma (ESCC) is a common type of esophageal cancer in Asia [2] while adenocarcinoma is the main type in western countries [2]. Treatment for esophageal cancer includes chemotherapy, radiation therapy, and surgery; postoperative chemotherapy is considered to be an effective method for the prevention of postoperative recurrence [3]. Due to the lack of early symptoms, esophageal cancer is mostly advanced by the time it is diagnosed, which leads to a poor survival rate after esophagectomy [4]. A study has shown that the 5-year survival rate after three esophagectomies is only about 50% [5]. Although advances in diagnosis, staging, and therapy methods have slightly improved the survival rate of patients in recent years, esophageal cancer is still one of the most fatal cancers in the world.

As one of the main active ingredients of ginseng, ginsenoside has the functions of improving immunity [6], cardiovascular function [7,8], and memory [9] and also displays anti-aging [10] and anti-tumor properties. Ginsenoside Rh2 (G-Rh2) is a rare ginsenoside isolated during the processing of red ginseng, which can be divided into (20S) G-Rh2 and (20R) G-Rh2 according to the sheerness of its dammarane skeleton [11]. Studies have shown that (20S) G-Rh2 can inhibit the NF-κB signal pathway [12], inhibit cell growth and metastasis [13], and activate both intrinsic (mitochondria-mediated) and extrinsic (death receptor-related) apoptosis in a series of tumor cells [14,15]. Additionally, (20S) G-Rh2 significantly inhibited lymph node metastasis and tumor growth in colorectal cancer (LMN-CRC) in vivo [16]. Furthermore, hematoxylin-eosin (HE) staining showed that (20S) G-Rh2 did not inflict any heart, liver, spleen, lung, or kidney damage in the mice [16]. Thus, (20S) G-Rh2 is considered a promising natural anti-cancer compound.

(20S) G-Rh2 induces apoptosis in a variety of tumor cells, including human leukemia cells, human cervical cancer cells, human lung cancer cells, and human ovarian cancer cells [17], but its effect on esophageal cancer cells is still unclear. In this research, we studied the effect of (20S) G-Rh2 in the highly differentiated esophageal squamous cell ECA109 and poorly differentiated esophageal squamous cell TE-13 and demonstrated that (20S) G-Rh2 can inhibit cell growth in both ECA109 and TE-13 cells by inducing apoptosis with different mechanisms.

## 2. Results

### 2.1. (20S) G-Rh2 Inhibits the Cell Growth of ECA109 and TE-13 Cells

**Figure 1:**
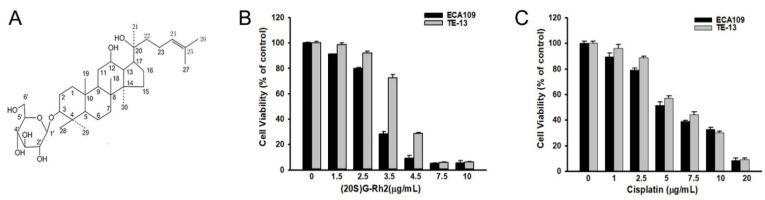
(**A**) Chemical structure of (20S) ginsenoside Rh2; (**B**) cell viability assay of ECA109 and TE-13 cells treated with (20S) ginsenoside Rh2. (20S) G-Rh2 showed cell growth inhibition in ECA109 and TE-13 cells with an IC50 of 2.9 and 3.7 μg/mL, respectively; (**C**) cell viability assay of ECA109 and TE-13 cells treated with Cisplatin. Cisplatin showed cell growth inhibition in ECA109 and TE-13 cells with an IC50 of 5.7 and 6.3 μg/mL, respectively.

We first tested the effect of (20S) G-Rh2 on the cell viability of esophageal cancer cells with a cell viability assay. The results showed that (20S) G-Rh2 has a strong cell growth inhibitory effect in both ECA109 and TE-13 cells, with an IC50 of 2.9 and 3.7 μg/mL, respectively (Figure 1). Cisplatin was used as a positive drug.

### 2.2. (20S) G-Rh2 Inhibits the Proliferation of Esophageal Cancer Cells by Inducing Apoptosis

**Figure 2:**
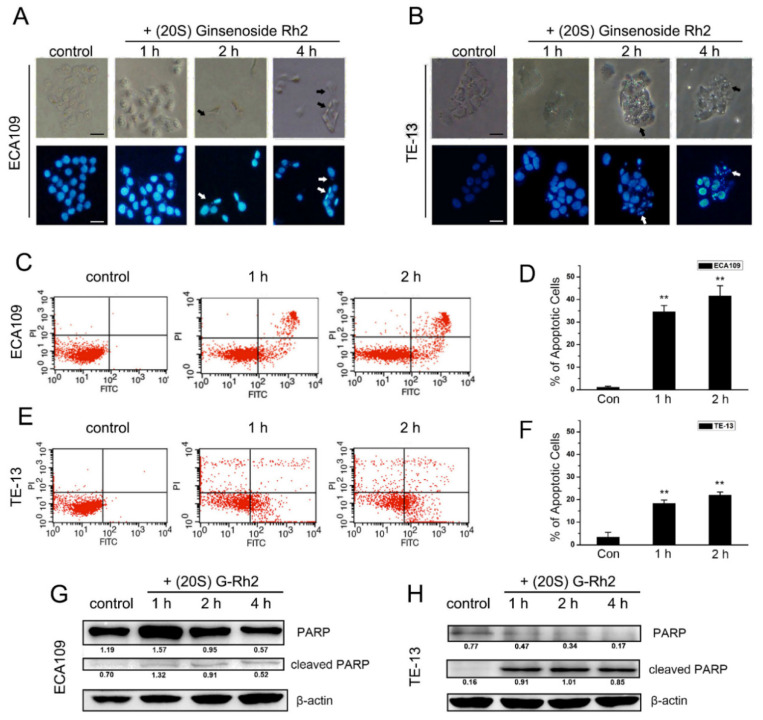
(20S) ginsenoside Rh2-induced apoptosis in human esophageal cells. (**A**,**B**) ECA109 and TE-13 cells were treated with 7.5 μg/mL (20S) G-Rh2 for 1, 2, and 4 h. Cells were stained with DAPI and photographed by a fluorescence microscope. Scale bar: 20 μm; (**C**–**F**) cells were stained with Annexin V-FITC/PI and analyzed by flow cytometry after being treated with 7.5 μg/mL G-Rh2. Cells at the early stage of apoptosis were positive for Annexin V-FITC and negative for PI. Cells that were positive for both Annexin V-FITC and PI were at the end stage of apoptosis; (**G**,**H**) as the substrate of Caspase-3, cleaved PARP was identified by Western blotting. ** *p* < 0.01.

In order to prove that (20S) G-Rh2 inhibits esophageal cancer cell proliferation by inducing apoptosis, ECA109 and TE-13 cells were treated with (20S) G-Rh2, and both of them showed significant morphological characteristics of apoptosis (Figure 2A,B). Then, we studied the apoptotic ratio after (20S) G-Rh2 treatment by flow cytometry. The results showed that after treatment with 7.5 μg/mL (20S) G-Rh2 for 1 h, the apoptotic ratio of ECA109 and TE-13 cells were 34.59% and 18.29%, respectively, which would be 41.64% and 21.97% when the duration of (20S) G-Rh2 treatment was extended to 2 h (Figure 2C–F).

**Figure 3:**
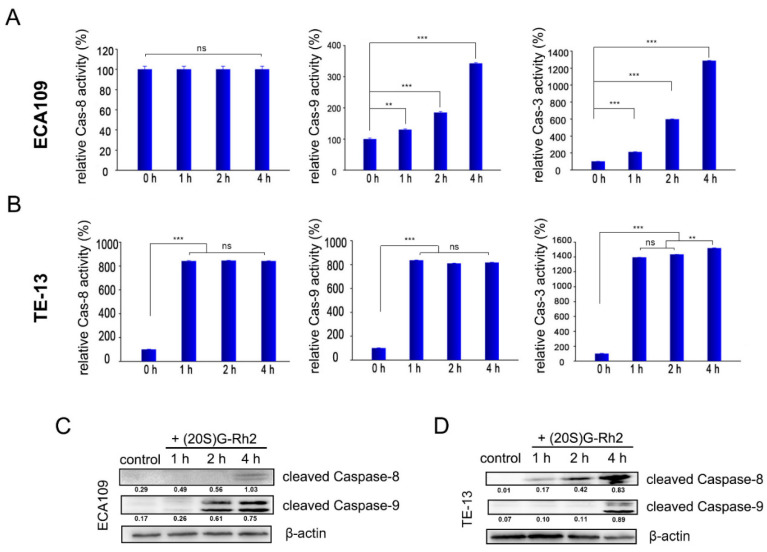
(**A**,**B**) Activity assay of Caspase-8, Caspase-9, and Caspase-3 in ECA109 and TE-13 cells. Cells were treated with 7.5 μg/mL G-Rh2 for the indicated times. Cell-free Caspase-8, -9, and -3 activities were detected by using specific substrates: Ac-IETD-AFC (for Caspase-8), Ac-LEHD-AFC (for Caspase-9), and Ac-DEVD-AFC (for Caspase-3); (**C**,**D**) Western blot results of cleaved Caspase-8 and cleaved Caspase-9 in ECA109 and TE-13 cells after being treated with (20S) G-Rh2. ** *p* < 0.01, *** *p* < 0.001.

We also examined the activity of Caspase-3 in ECA109 and TE-13 cells, which were treated with 7.5 μg/mL (20S) G-Rh2 for 1, 2, and 4 h. Caspase-3 activity in ECA109 and TE-13 cells both time-dependently increased, which increased 11-fold to the control after 4 h in ECA109 and increased 12-fold to the control after only 1 h in TE-13 (Figure 3A,B). As the substrate of Caspase-3, cleaved PARP in both ECA109 and TE-13 cells was also examined by Western blotting (Figure 2G,H). These results demonstrated that (20S) G-Rh2 inhibits the growth of ECA109 and TE-13 cells by inducing apoptosis.

### 2.3. (20S) G-Rh2 Activates the Intrinsic Apoptosis Pathway in Esophageal Cancer Cells

We first tested the activation of the intrinsic apoptosis pathway in ECA109 and TE-13 cells. Cells were treated with 7.5 μg/mL (20S) G-Rh2 for 1, 2, and 4 h; then, Caspase-9 activity in the whole lysis was tested by using an Ac-LEHD-AFC kit. We found that the activity of Caspase-9 was time-dependent and increased in both ECA109 and TE-13 cells, and these results were consistent with the results of Western blotting assay (Figure 3).

**Figure 4:**
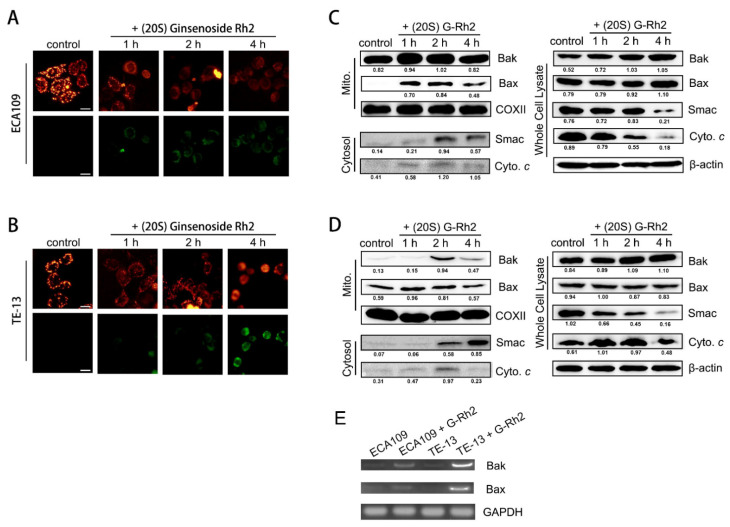
(20S) ginsenoside Rh2-induced intrinsic apoptosis in human esophageal cells. (**A**,**B**) (20S) G-Rh2 induces both mitochondrial membrane depolarization in ECA109 and TE-13 cells. Cells were treated with 7.5 μg/mL (20S) G-Rh2 for 1, 2, and 4 h and strained with the mitochondrial membrane potential assay kit with JC-1. Images were captured by using a fluorescence microscope at the excitation wavelengths of 570 and 500 nm; (**C**,**D**) apoptosis regulated proteins in cytosolic and mitochondrial fractions as well as in the total cell lysates that were analyzed by Western blotting; (**E**) RT-PCR results of Bax and Bak upon treatment with (20S) G-Rh2. Both Bax and Bak were upregulated in mRNA levels in ECA109 and TE-13 cells.

**Figure 5:**
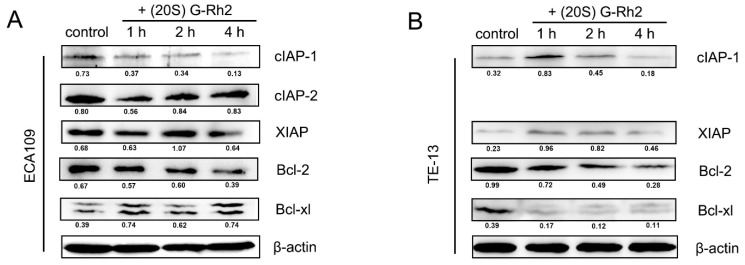
The expression level of apoptosis-regulated proteins in ECA109 (**A**) and TE-13 (**B**) cells after being treated with (20S) G-Rh2.

The decrease of the mitochondrial membrane potential is an early event of intrinsic apoptosis and is closely related to the release of Smac and cytochrome *c*. In our study, the mitochondrial membrane potential was detected by using a mitochondrial membrane potential assay kit with JC-1. Briefly, both ECA109 and TE-13 cells were treated with 7.5 μg/mL (20S) G-Rh2 and a JC-1 buffer for 4 h; then, the fluorescence of the JC-1 monomer (green) and aggregates (red in the matrix) were observed by a fluorescence microscope. The red dot-shaped fluorescence on mitochondria gradually diffused, and the green fluorescence increased in both ECA109 and TE-13, indicating the decrease of the mitochondrial membrane potential (Figure 4A,B). The RT-PCR and Western blotting assay showed upregulated expression levels and mitochondrial localization of Bax and Bak while Smac and cytochrome *c* translocated from the mitochondria to the cytoplasm (Figure 4C–E). The expression level of anti-apoptotic proteins was also examined and showed that cIAP-1 and Bcl-2 were downregulated after being treated with (20S) G-Rh2 (Figure 5). Taken together, it can be concluded that (20S) G-Rh2 induced apoptosis in esophageal cancer cells by activating the mitochondria-mediated intrinsic apoptosis pathway. Figure S1 shows this in detail: (20S) G-Rh2-induced caspase-dependent apoptosis in esophageal cancer cells identified by cell viability assay.

### 2.4. (20S) G-Rh2 Activates the Extrinsic Apoptosis Pathway of TE-13 Cells

**Figure 6:**
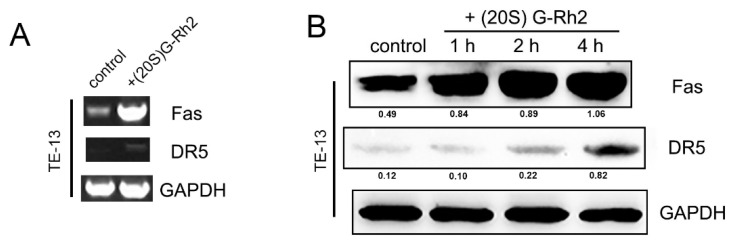
Changes at mRNA and protein levels of the death receptor family members in TE-13 cells after being treated with (20S) G-Rh2. (**A**) RT-PCR results of TE-13 cells after being treated with (20S) G-Rh2; (**B**) protein levels of death receptors Fas and DR5 in TE-13 cells after being treated with (20S) G-Rh2.

The death receptor signal pathway is another important pathway to induce apoptosis. In our research, we also tested the changes of Caspase-8 activity. We found that after treating with (20S) G-Rh2 for 1 h, Caspase-8 activity in TE-13 increased 7-fold to the control group but did not change in ECA109 (Figure 3A,B). The result of Western blotting also showed a cleaved Caspase-8 band in the TE-13 cells, which deepened time-dependently (Figure 3C,D). These results indicated that (20S) G-Rh2 could activate the death receptor apoptosis pathway in TE-13 cells. Subsequently, we investigated the transcription and expression levels of a series of death receptor family members in TE-13 cells by using RT-PCR and Western blotting. The results showed that death receptors Fas and DR5 were significantly upregulated at both the protein and mRNA levels (Figure 6A,B). Therefore, we speculated that (20S) G-Rh2 can activate the extrinsic apoptosis pathway in TE-13 cells by upregulating the transcription and expression levels of death receptor family members.

Regulating the expression level of Fas and DR5 is associated with interactions between multiple transcription factors. We investigated the transcription factors regulating the expression of Fas and DR5 genes in the GeneCards database and found there are 167 transcription factors regulating Fas and 272 transcription factors regulating DR5. In a transcriptome analysis of TE-13 cells conducted by our research group, we found that 45 of the 167 transcription factors regulating Fas (such as EGR1, ATF3, JUN, and MAFF) and 68 of the 272 transcription factors regulating DR5 (such as ATF3, NR4A1, MAFF, and NFIL3) were upregulated at the mRNA level after being treated with 7.5 μg/mL (20S) G-Rh2 for 4 h. Transcription factors that significantly upregulated at the mRNA level after the (20S) G-Rh2 treatment in the TE-13 cell lines are shown in Tables S2 and S3. These results suggest that (20S) G-Rh2 may regulate the expression of Fas and DR5 genes by targeting several transcription factors. Table S2: list of upregulated TFs with the regulating Fas gene; Table S3: list of upregulated TFs with the regulating DR5 gene.

## 3. Discussion

Apoptosis plays an important role in the growth and development of multicellular organisms. Excessive apoptosis may lead to degenerative disease while insufficient apoptosis may lead to tumor disease. In cancer cells, dysregulation of apoptosis caused by blocked apoptotic signaling pathways often occurs, and cancer cells gain an excessive survival advantage by disabling apoptotic mechanisms [18]. An apoptosis pathway can be divided into intrinsic apoptosis and extrinsic apoptosis pathways; the former is mitochondria-centered and eventually activates Caspase-3 and Caspase-7 by activating Caspase-9 while the latter starts from the binding of death receptors (members of the TNF receptor superfamily such as TNF-R1, Fas, DR3, DR4, and DR5) to their ligands (mainly members of the TNF family) and then activates Caspase-8 via DISC (death-inducing signaling complex) and eventually activates Caspase-3. Activated Caspase-8 can also transduce apoptotic signals to mitochondria through cascade amplification and then trigger intrinsic apoptotic pathways [19]. In the result of Western blotting, we found that the band of cleaved Caspase-8 in TE-13 cells appeared earlier than the band of cleaved Caspase-9, suggesting that the extrinsic apoptosis pathway was activated first in TE-13 cells and promoted the activation of the intrinsic apoptosis pathway. Both Caspase-8 and Caspase-9 were activated in TE-13 cells while only intrinsic apoptotic pathways were activated in ECA109 cells, which may be the reason for the slower increase of Caspase-3 activity in ECA109 cells.

The response of apoptosis pathways to multiple physiological signals and cytotoxic factors is controlled by Bcl-2 family proteins [20,21]. Oligomeric Bax and Bak destroys the integrity of the mitochondrial outer membrane and releases Smac and cytochrome c to the cytoplasm [22] while Bcl-2 inhibits the mitochondrial localization of Bax and Bak and prevents the release of these pro-apoptotic proteins [23]. In this study, both ECA109 and TE-13 cells showed time-dependent downregulation of Bcl-2 protein levels and activation of intrinsic apoptosis pathways. At the same time, it was also observed that the expression level of Bcl-xl protein was downregulated in TE-13 cells, in which the activity of Caspase-9 was increased more rapidly than in ECA109 cells.

(20S) G-Rh2 has been shown to induce apoptosis in a variety of cancer cell lines by activating mitochondrial or death receptor-mediated apoptosis pathways [14]. Both in vitro and in vivo experiments have shown that (20S) G-Rh2 can inhibit growth and metastasis of tumor cells, so it is considered a promising natural compound for the treatment of cancer. In a previous study, it was demonstrated that (20S) G-Rh2 can activate the extrinsic apoptosis pathway in a p53-dependent manner [24]. However, in this research, both the transcription and expression level of Fas and DR5 in TE-13 were upregulated after being treated with (20S) G-Rh2 although TE-13 is a cell line with dysfunctional p53, suggesting that (20S) G-Rh2 can also trigger the extrinsic apoptosis pathway in a non-p53-dependent way. This result may be related to the fact that (20S) G-Rh2 has multiple intracellular targets and regulates the activity of several transcription factors. These specific mechanism remains need to be studied in future.

## 4. Materials and Methods

### 4.1. Materials

ECA109 and TE-13 cell lines were from the Affiliated Cancer Hospital and the Institute of Guangzhou Medical University. The (20S) G-Rh2 (≥97%) was purchased from Sigma Aldrich and dissolved in 75% ethanol at a concentration of 7.5 mg/mL and stored at −80 °C. The Cisplantin (≥98%) was purchased from Sigma Aldrich and dissolved in a phosphate buffer saline (PBS) at a concentration of 10 mg/mL and stored at −80 °C. Caspase substrates, Ac-DEVD-AFC, Ac-IETD-AFC, and Ac-LEHD-AFC were purchased from Calbiochem (La Jolla, CA, USA). The Mitochondria Isolation Kit was purchased from Pierce (Rockford, IL, USA). The mitochondrial membrane potential assay kit with JC-1 was purchased from Beyotime (Shanghai, China). Antibodies to Smac, Bax, Bak, PARP, Bcl-xL, cIAP-1, cIAP-2, and β-Actin were purchased from SantaCruz Biotechnology (Santa Cruz, CA, USA). Antibodies to Caspase-8, Caspase-9, cytochrome *c*, XIAP, and COXII were purchased from Cell Signaling Technology (Beverly, MA, USA).

### 4.2. Cell Culture

ECA109 and TE-13 cells were cultivated in Dulbecco’s Modified Eagle’s Medium (DMEM) with 10% newborn calf serum at 37 °C in a CO_2_ incubator with 5% CO_2_. The culture medium contained 100 μg/mL of penicillin and 100 μg/mL of streptomycin_._

### 4.3. Cell Viability Assay

Exponentially growing ECA109 and TE-13 cells were resuspended in DMEM and seeded into a 96-well plate at 1 × 10^4^ cells per well. After 24 h of incubation at 37 °C with 5% CO_2_, cells were treated with increasing concentrations of (20S) G-Rh2 (1.5, 2.5, 3.5, 4.5, 7.5, 10 μg/mL) for 48 h. The Cisplan (1, 2.5, 5, 7.5, 10, 20 μg/mL) was used as a positive control under the same conditions. At the end of the treatment, 20 μL 3-(4, 5-dimethylthiazol-2-yl-2,5-diphenyltetrazoniumbromide (MTT) (5 mg/mL) was added to each well, followed by an additional 4 h of incubation. The formazan grains formed by viable cells were solubilized with 200 μL DMSO, and the color intensity was measured at 550 nm with an ELISA reader (BioTek Instruments, Winooski, VT, USA).

### 4.4. Apoptosis Analysis

Cells were treated with 7.5 μg/mL (20S) G-Rh2 in fresh serum-free DMEM for 1, 2, and 4 h. Then cells were fixed in 70% ethanol at −20 °C for 15 min and stained with 1 μg/mL DAPI. Fluorescent cells were photographed under a fluorescence microscope (Olympus, Tokyo, Japan)

### 4.5. Western Blotting Assay

Esophageal cells were treated with (20S) G-Rh2. The treated cells were washed with ice-cold PBS twice and solubilized in a lysis buffer containing 20 mM Tris pH 7.5, 2 mM MgCl_2_, 1 mM DTT, 0.5% Triton X-100, 1 mM EGTA, 25 mM NaF, 1 mM Na_3_VO_4_, 50 mM glycerol phosphate, 2 mg/mL leupeptin, 2 mg/mL pepstatin A, 2 mg/mL antipain, and 1 mM PMSF. After incubating on ice for 1 h, the insoluble materials were removed by centrifugation at 12,000× *g* for 15 min. The equal amount of cell lysate was separated by SDS-PAGE and electrotransferred onto a fixed PVDF membrane. The membrane was blocked with 5% skim milk in PBST (PBS with 0.1% *v/v* Tween 20) for 1 h at RT and then incubated with antibodies at 4 °C overnight. Then, membranes were washed with PBST and incubated with a horseradish peroxidase-coupled, anti-mouse immunoglobulin G (IgG) or an anti-rabbit IgG secondary antibody (Pierce) for 1 h at RT and then detected with an electrogenerated chemiluminescence (ECL) revelation system (Tanon, Shanghai, China). The relative intensity of the Western blotting assay was calculated by using Image J and displayed under each band. The absolute gray value of the target strip was calculated and then divided by the absolute gray value of its corresponding internal reference (β-actin for proteins in the cytoplasm and whole cell lysate; COXII to obtain the relative intensity for proteins in the mitochondria).

### 4.6. Flow Cytometry and Annexin V Assay

1 × 10^6^ exponentially growing esophageal cells were harvested and resuspended with a 400 μL binding buffer in an Annexin V Assay Kit. A total of 200 μL of the cells were incubated with a positive control buffer for 10 min at RT, washed with PBS, and resuspended with the 200 μL binding buffer again. Then, cells were mixed again and separated into 3 parts (130 μL per tube) and added to the 5 μL Annexin V-FITC, PI solution, or PBS and incubated in the dark for 10 min at RT as the control.

Esophageal cells were treated with 7.5 μg/mL (20S) G-Rh2 for 1 or 2 h and then harvested. Cells were washed with PBS and resuspended with a 1 mL binding buffer. A total of 100 μL of the cells were transferred into a new tube and incubated for 10 min in the dark with the 5 μL Annexin V-FITC and then incubated for 5 min with the 5 μL PI solution and added PBS up to 500 μL. The percentage of Annexin V(+) cells was determined by flow cytometry, which indicates the frequency of the total apoptotic cells (Becton Dickinson FACS Calibur Cytometer).

### 4.7. Caspase Activity Assay

Esophageal cells (1.6 × 10^6^) were treated with 7.5 μg/mL (20S) G-Rh2 for 1, 2, and 4 h before harvested. Cell lysates was incubated with 200 mM Ac-DEVD-AFC (for Caspase-3), Ac-IETD-AFC (for Caspase-8), and Ac-LEHD-AFC (for Caspase-9) in a reaction buffer containing 20 mM HEPES, pH 7.4, 100 mM NaCl, 10 mM DTT, 0.1% CHAPS, and 10% sucrose at 37 °C for 1 h. The activity of Caspase-8, Caspase-9, and Caspase-3 was analyzed by measuring the absorption of the cell lysates at 405 nm after incubation with Caspase substrates, using a microplate reader (TECAN, Maennedorf, Switzerland).

### 4.8. RT-PCR

The sequences of primers used in the RT-PCR assay are in Table S1. Cells were suspended by Trizol (Life Technologies Corporation, California, CA, USA) to exact RNA after harvest. Reverse transcription (RT) was performed using the StarScript II First-strand cDNA Synthesis Kit-II (GenStar, Beijing, China). The RT mixture contained 1μg template RNA, 1 μL oligo (dT)_18_, 10 μL reaction mix, 1μL StarScript II RT mix, and 8 μL H_2_O. The reaction mixture was incubated in a thermocycler programmed at 50 °C for 40 min and then heated at 85 °C for 5 min to denature the StarScript II RT mix.

### 4.9. RNA-Seq Analysis

5 × 10^6^ TE-13 cells in a 6-well plate were harvested in 1 mL Trizol and were used for global transcriptome analysis by Annoroad Gene Technology (Beijing, China). Significant differentially expressed genes between the (20S) G-Rh2-treated and control groups were identified with the cutoff of *p* < 0.05 and |Log2FoldChange| > 2.

### 4.10. Statistical Analysis

Data were presented as a mean ± standard deviation with Microsoft Office 2013. The statistical significance was calculated with the Student’s *t*-test. Differences were considered statistically significant as follows: * *p* < 0.05; ** *p* < 0.01; *** *p* < 0.001.

## Data Availability

The data presented in this study are available in the Supplementary Material.

## Supplementary Materials

The following supporting information can be downloaded at https://www.mdpi.com/article/10.3390/molecules27175602/s1. Figure S1: (20S) G-Rh2-induced caspase-dependent apoptosis in esophageal cancer cells identified by cell viability assay; Table S1: primers used in this study; Table S2: list of upregulated TFs with the regulating Fas gene; Table S3: list of upregulated TFs with the regulating DR5 gene.

## Sample Availability

Samples of the compounds are not available from the authors.

## References

[1] Lee H.S., Park S.Y., Jang H.J., Kim M.S., Lee J.M., Zo J.I. (2012). Free jejunal graft for esophageal reconstruction using end-to-side vascular anastomosis and extended pharyngo-jejunostomy. Ann. Thorac. Surg..

[2] Wang B., Ge Y., Gu X. (2016). Analysis of esophageal cancer cell lines exposed to X-ray based on radiosensitivity influence by tumor necrosis factor-α. J. X-Ray Sci. Technol..

[3] Akiyama H., Tsurumaru M., Udagawa H., Kajiyama Y. (1994). Radical lymph node dissection for cancer of the thoracic esophagus. Ann. Surg..

[4] Nakajima M., Kato H., Miyazaki T., Kuwano H. (2011). ‘Guidelines for diagnosis and treatment of carcinoma of the esophagus’ revised version—The comparison to western countries. Nihon Rinsho.

[5] Kato H., Watanabe H., Tachimori Y., Iizuka T. (1991). Evaluation of neck lymph node dissection for thoracic esophageal carcinoma. Ann. Thorac. Surg..

[6] Tran T.L., Kim Y.R., Yang J.L., Oh D.R., Dao T.T., Oh W.K. (2014). Dammarane triterpenes from the leaves of panax ginseng enhance cellular immunity. Bioorg. Med. Chem..

[7] Zhang G., Liu A., Zhou Y., San X., Jin T., Jin Y. (2008). Panax ginseng ginsenoside-Rg2 protects memory impairment via anti-apoptosis in a rat model with vascular dementia. J. Ethnopharmacol..

[8] Li-Ping Z., Yi-Chuan J., Xiao-Feng Y., Hua-Li X., Min L., Xue-Zhong Z. (2016). Ginsenoside rg3 improves cardiac function after myocardial ischemia/reperfusion via attenuating apoptosis and inflammation. Evid Based Complement Altern. Med..

[9] Wang Z.L., Chen L.B., Qiu Z., Chen X.B., Liu Y., Li J., Wang L., Wang Y.P. (2018). Ginsenoside Rg1 ameliorates testicular senescence changes in D-gal-induced aging mice via anti-inflammatory and antioxidative mechanisms. Mol. Med. Rep..

[10] Zhou Y., Liu J., Cai S., Liu D., Jiang R., Wang Y. (2015). Protective effects of ginsenoside Rg1 on aging Sca-1^+^ hematopoietic cells. Mol. Med. Rep..

[11] Odashima S., Ohta T., Kohno H., Matsuda T., Kitagawa I., Abe H., Arichi S. (1985). Control of phenotypic expression of cultured B16 melanoma cells by plant glycosides. Cancer Res..

[12] Wang Y.S., Lin Y., Li H., Li Y., Song Z., Jin Y.H. (2017). The identification of molecular target of (20S) ginsenoside Rh2 for its anti-cancer activity. Sci. Rep..

[13] Wang Y.S., Li H., Li Y., Zhang S., Jin Y.H. (2020). (20S)G-Rh2 Inhibits NF-κB Regulated Epithelial-Mesenchymal Transition by Targeting Annexin A2. Biomolecules.

[14] Song B.K., Kim K.M., Choi K.D., Im W.T. (2017). Production of the Rare Ginsenoside Rh2-MIX (20(*S*)-Rh2, 20(*R*)-Rh2, Rk2, and Rh3) by Enzymatic Conversion Combined with Acid Treatment and Evaluation of Its Anti-Cancer Activity. J. Microbiol. Biotechnol..

[15] Chen Y., Liu Z.H., Xia J., Li X.P., Li K.Q., Xiong W., Li J., Chen D.L. (2006). 20(S)-ginsenoside Rh2 inhibits the proliferation and induces the apoptosis of KG-1a cells through the Wnt/β-catenin signaling pathway. Oncol. Rep..

[16] Yuan Y.H., Wang J., Xu M., Zhang Y.P., Wang Z.Q., Liang L.L., Sun P. (2020). 20(S)-ginsenoside Rh2 as agent for the treatment of LMN-CRC via regulating epithelial–mesenchymal transition. Biosci. Rep..

[17] He B., Chen P., Yang J., Yun Y., Zhang X., Yang R., Shen Z. (2012). Neuroprotective effect of 20(R)-ginsenoside Rg(3) against transient focal cerebral ischemia in rats. Neurosci. Lett..

[18] Alves N.L., Derks I.A., Berk E., Spijker R., van Lier R.A., Eldering E. (2006). The Noxa/Mcl-1 axis regulates susceptibility to apoptosis under glucose limitation in dividing T cells. Immunity.

[19] Matsuura K., Canfield K., Feng W., Kurokawa M. (2016). Metabolic Regulation of Apoptosis in Cancer. Int. Rev. Cell Mol. Biol..

[20] Thorburn A. (2004). Death receptor-induced cell killing. Cell Signal..

[21] Nagasaka A., Kawane K., Yoshida H., Nagata S. (2010). Apaf-1-independent programmed cell death in mouse development. Cell Death Differ..

[22] Wei M.C., Zong W.X., Cheng E.H., Lindsten T., Panoutsakopoulou V., Ross A.J., Roth K.A., MacGregor G.R., Thompson C.B., Korsmeyer S.J. (2001). Proapoptotic BAX and BAK: A requisite gateway to mitochondrial dysfunction and death. Science.

[23] Fujita T., Terada S., Ueda H., Suzuki E. (1996). Overexpression of bcl-2 improved survival of cos-1 cells and enhanced transient protein production. J. Ferment. Bioeng..

[24] Guo X.X., Guo Q., Li Y., Lee S.K., Wei X.N., Jin Y.H. (2012). Ginsenoside Rh2 induces human hepatoma cell apoptosisvia bax/bak triggered cytochrome C release and Caspase-9/Caspase-8 activation. Int. J. Mol. Sci..

