# Supplementary material for: (20S) Ginsenoside Rh2-Activated, Distinct Apoptosis Pathways in Highly and Poorly Differentiated Human Esophageal Cancer Cells
Source: Molecules. 2022 Aug 31;27(17):5602. doi: 10.3390/molecules27175602 (PMC9457866; doi:10.3390/molecules27175602)
Supplement: Supplementary file 1 [file molecules-27-05602-s001.zip › Figure S1& Table S1.pdf]

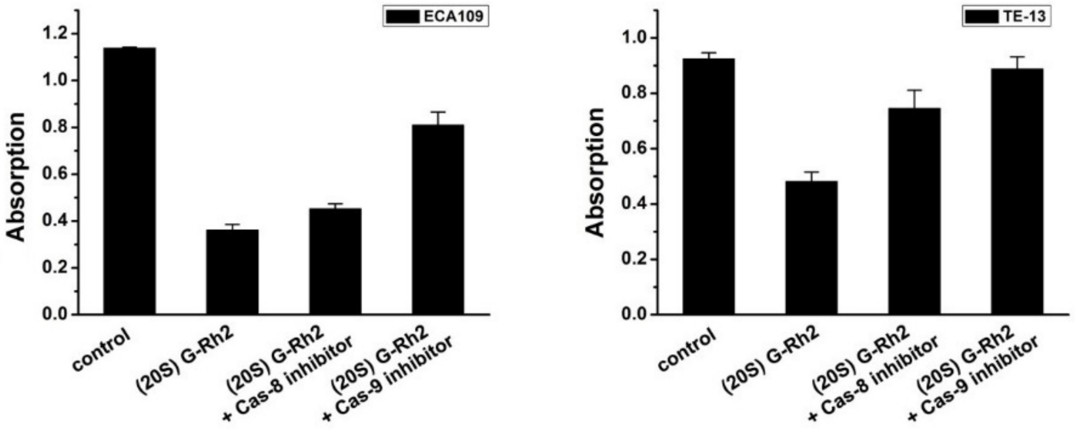

Figure S1. (20S)G-Rh2 induced caspase-dependent apoptosis in esophageal cancer cells identified by cell viability assay. ECA109 and TE-13 cells were co-treated with 5μg/mL (20S)G-Rh2 and 20μM inhibitor of caspase-8(Z-IEHD-FMK, MCE) or caspase-9 (Z-LETD-FMK, MCE) for 48 hours in a 96-wells plate. After treated with MTT, cells were solubilized with DMSO and measured at 550nm with an ELISA reader.

Table S1. Primers used in this study

Gene	Primers Sequences
Bax_F	TCAGGATGCGTCCACCAAGAAG
Bax_R	TGTGTCCACGGCGGCAATCATC
Bak_F	TTACCGCCATCAGCAGGAACAG
Bak_R	GGAACTCTGAGTCATAGCGTCG
Fas_F	ATGCTGGGCATCTGGACCCTC
Fas_R	CTAGACCAAGCTTTGGATTTCATTTCTG
DR5_F	ATGGAACAACGGGGACAGAAGG
DR5_R	TTAGGACATGGCAGAGTCTGCATTAC
GAPDH_F	ATGGGGAAGGTGAAGGTCGGAG
GAPDH_R	TTACTCCTTGGAGGCCATGTGGGCC
